# A Rare Cause of Medial Thigh Pain: Does Point-of-Care Ultrasound Scan Have a Role in the Diagnosis of Obturator Hernia in the Emergency Room?

**DOI:** 10.7759/cureus.33547

**Published:** 2023-01-09

**Authors:** Yakub W Ibrahim, Philip Tabi, Abdurrahman Yusuf

**Affiliations:** 1 Emergency Medicine, Mid and South Essex University Hospital NHS Trust, Southend-On-Sea, GBR

**Keywords:** thigh pain, accident and emergency, medial thigh pain, emergency room (er), point-of-care ultrasound (pocus), obturator hernia

## Abstract

Obturator hernia describes the protrusion of the intra-abdominal viscus through the obturator foramen. It accounts for 0.05-1.4% of all hernias. We present a case of a 72-year-old female, with right-sided inner thigh pain of sudden onset who was subsequently diagnosed with an obturator hernia in the emergency room with point-of-care ultrasound (POCUS). She subsequently had laparoscopic surgery and improved without complications.

## Introduction

Obturator hernia describes the protrusion of the intra-abdominal viscus through the obturator foramen. It would often manifest without triggering events with clinical features that could mimic other common conditions affecting the lower extremities [1]. Though obturator hernia is a rare condition, accounting for 0.05-1.4% of all hernias, missed diagnosis has been noted to be associated with increased mortality [1]. It is increasingly being diagnosed in patients presenting with features of intestinal obstruction [2,3].

## Case presentation

A 72-year-old female presented to the emergency room with a three-hour history of sudden onset right-sided inner thigh pain without any precipitating event. She was unable to lift the right leg or weight bear due to the associated excruciating pain which she had attributed to her hip replacement. She denies a history of swelling, redness, or warmth. No history is suggestive of fever or trauma to the limb.

She reports nausea but denies any history of abdominal distention, pain, or vomiting, and had passed flatus on the day of the presentation. She last moved her bowels the day before the presentation. Her pain persisted despite analgesia administered by the Paramedics en route to the hospital. However, the pain intensity was reduced with morphine administered while in the emergency room.

Her medical history includes a right hip osteoporotic fracture five years prior to presentation with a prosthetic hip replacement and hypertension. The review of systems was unremarkable.

On examination, her vital signs were within normal limits, she was afebrile and well-hydrated with no pedal edema.

Examination of the lower limbs revealed tenderness on the medial aspect of the right thigh which was reproducible on internal rotation of the right leg. No obvious deformity, swelling, no differential warmth, or erythema. The abdomen was non-tender, not distended with normal bowel sound, and examination of the inguinal and the femoral regions was normal. Examination of the chest and cardiovascular systems was unremarkable.

An initial impression of prosthetic hip dislocation was entertained, X-ray of the hips and pelvis showed a normal right hip prosthesis.

**Figure 1:**
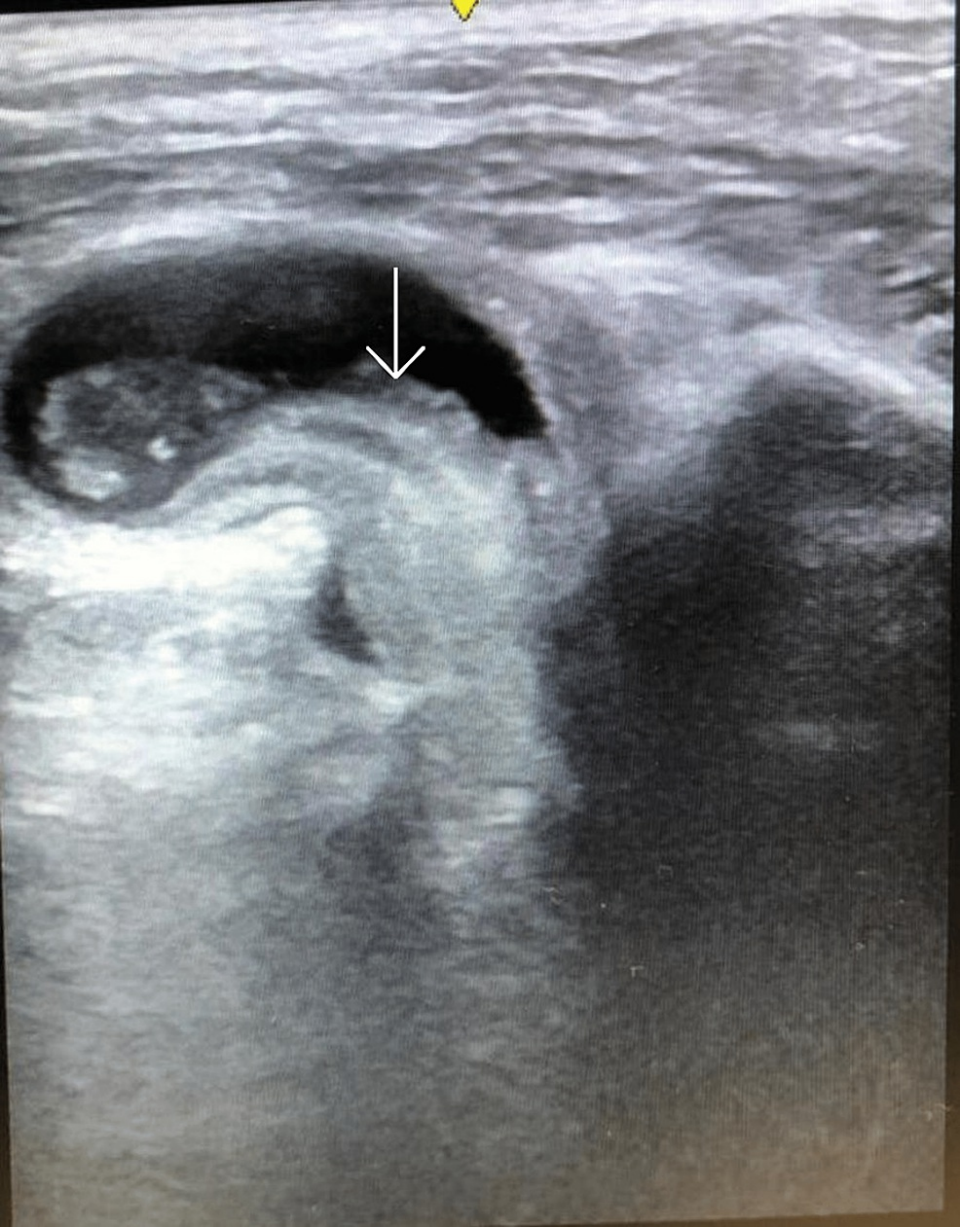
POCUS scan of the inguinal region (transverse plane) showing the obturator hernia with incarcerated loops of bowel (white arrow). POCUS: point-of-care ultrasound

**Figure 2:**
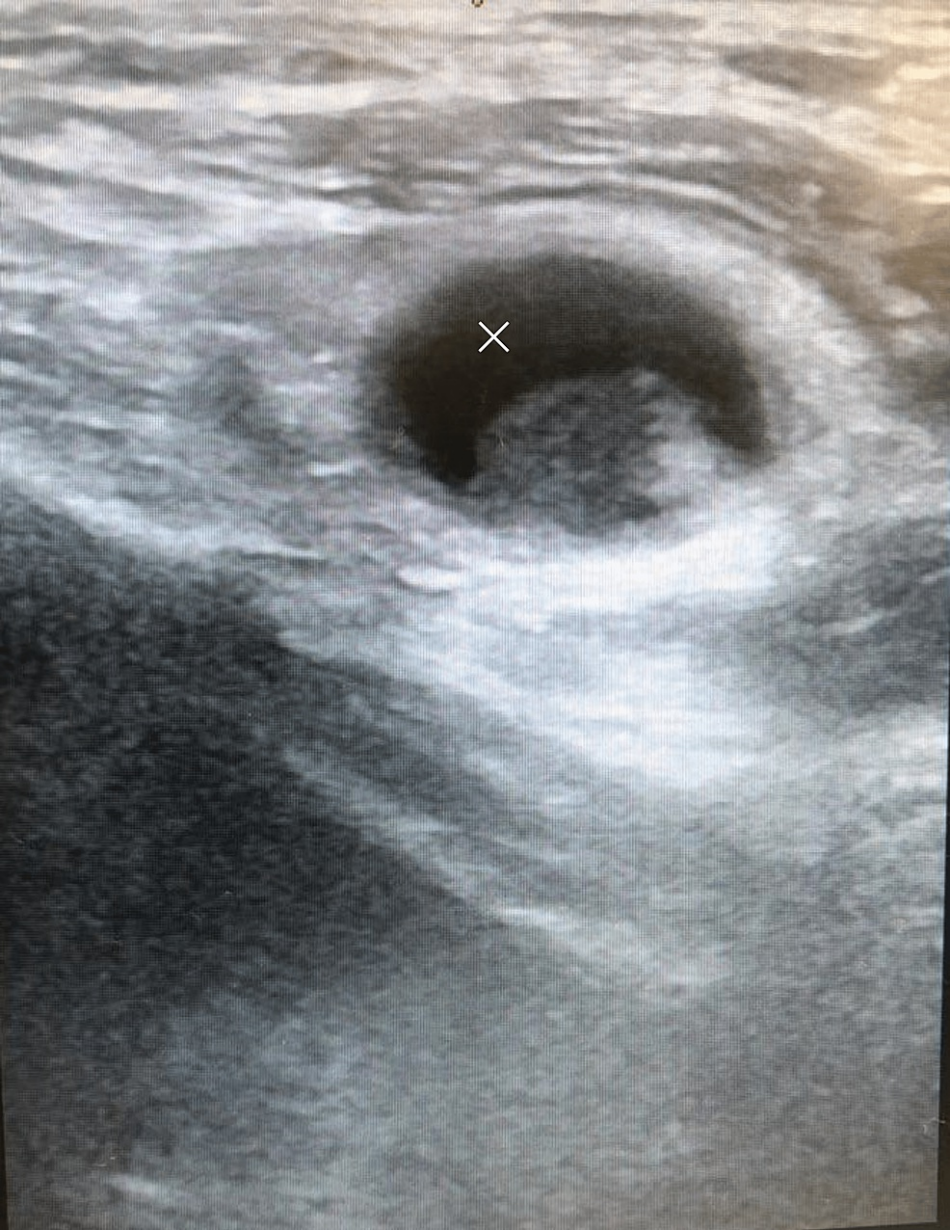
POCUS of the inguinal region showing the obturator hernia in the longitudinal plane (white star). POCUS: point-of-care ultrasound

Bedside point-of-care ultrasound (POCUS) of the right inguinal region performed in the emergency room using the linear probe revealed a hernia sac that had small bowel incarcerated with bowel edema (Figures 1 and 2).

**Figure 3:**
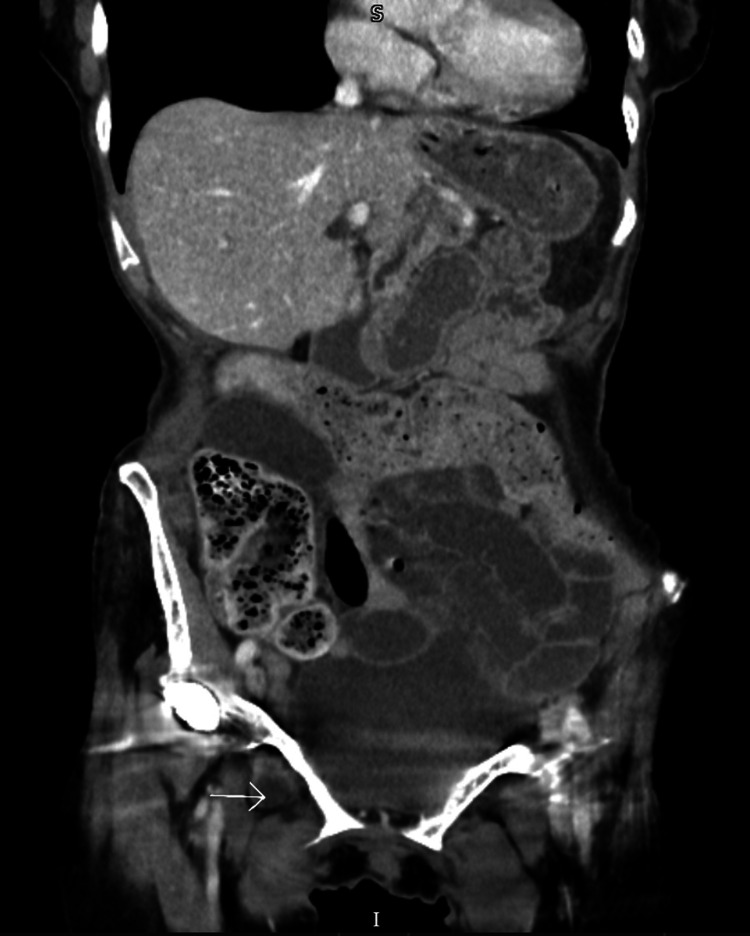
CT scan of abdomen and pelvis (coronal plane) showing the right-sided obturator hernia (arrow). CT: computed tomography

Eventually, a computer tomography (CT) scan of the abdomen and pelvis confirmed incarcerated right obturator hernia with small bowel obstruction (Figure 3).

The patient was kept nil by mouth, given IV antibiotics, and admitted to the surgical team. She had emergency laparoscopic surgery which was uneventful. There was no bowel necrosis and the patient recovered fully. The patient was subsequently discharged home after a four-day stay in the hospital.

## Discussion

This patient presented with right-sided thigh pain with no history of trauma or signs of infection and was subsequently diagnosed in the emergency department with an obturator hernia using POCUS.

As in our patient, obturator hernia would typically present with non-specific symptoms especially in lean elderly ladies, the findings of thigh pains in the anteromedial aspect often with no precipitating event, relieved by flexion and worsened by any other type of movement at the hip joint should raise the clinical suspicion for obturator hernia [4]. Though our patient did not present with clinical features of bowel obstruction, multiple reports have found that between 50% and 90% of patients diagnosed with obturator hernia already have bowel obstruction [5,6]. It affects females more than males with most patients noted to be elderly [7]. As in our patient, it would usually involve the right thigh more than the left particularly in females, though reports had found it affects both thighs equally in males [8].

Due to the deep location of the obturator foramen, the content of the obturator hernia may not be apparent on physical examination making the diagnosis difficult and easily missed [9].

Though a CT scan of the abdomen and pelvis continues to be the investigation of choice in the diagnosis of obturator hernia, as in our patient, the use of POCUS with a high-frequency probe in the emergency department, though user dependent, could help in the timely diagnosis and management of these patients thus reducing the morbidity and mortality associated with this condition [10]. This is even more important in resource-constraint settings where there could be delayed access to CT scans.

Ultrasound findings of an obturator hernia taken from the femoral triangle with a high-frequency probe may reveal a cystic mass behind the pectineus muscle in the longitudinal plane (this is would be differentiated from a femoral hernia which is anterior to the pectineus muscle). The peristaltic movement of the incarcerated bowel may be visualized depending on the extent and severity of the pathology [11].

While more research needs to be done to ascertain the sensitivity and specificity of POCUS in the diagnosis of obturator hernia, there has been documented use of POCUS in the diagnosis of obturator hernia [11,12].

Surgical intervention is often needed in the treatment of obturator hernia as most cases are diagnosed as bowel obstruction with incarceration [13].

## Conclusions

In conclusion, obturator hernia may not present with clinical features of bowel obstruction early in the disease process, it should be suspected particularly in lean elderly patients presenting with unprovoked medial thigh pain with no other cause noted. Delayed diagnosis is associated with increased morbidity and mortality. POCUS can prove key to timely diagnosis in the emergency room. More research into the use of POCUS in the diagnosis of obturator hernia is needed to facilitate its acceptance.
